# Four-Rooted Mandibular First Molar with an Unusual Developmental Root Fusion Line: A Case Report

**DOI:** 10.1155/2012/237302

**Published:** 2012-06-25

**Authors:** Jojo Kottoor, Denzil Valerian Albuquerque, Natanasabapathy Velmurugan, Mylswamy Sumitha

**Affiliations:** Department of Conservative Dentistry and Endodontics, Meenakshi Ammal Dental College, Chennai 600096, India

## Abstract

The paper describes the anatomical variation of four roots in a mandibular permanent first molar diagnosed using multiple angulated preoperative radiographs and its successful nonsurgical endodontic management. Careful observation and exploration of the pulpal floor using a dental operating microscope revealed a peculiar developmental root fusion line on the pulp chamber floor. Based on the above observation, a correlation between this unusual line and the existence of additional roots has been proposed and discussed.

## 1. Introduction

 One of the most important aspects in contemporary endodontics is a thorough knowledge of the internal and external root anatomy [1]. Additional roots and root canals if not detected could lead to endododontic failure [2]. Thus, a thorough knowledge of the root and root canal morphology and a good anticipation of their possible morphological variations will help reduce endodontic failure caused by incomplete debridement and obturation [3]. Anatomical variation is prevalent in many teeth, with some teeth having as many as 5 separate roots [4] or even seven [5, 6] or eight [7] separate root canals. The mandibular first molar, or the “6-year molar,” which is the largest tooth in volume and most complex in root and canal anatomy, is possibly the most treated and least understood posterior tooth [3]. The purpose of the present paper is to report the successful nonsurgical endodontic management of a four-rooted permanent mandibular first molar with each root containing its own independent root canal and additionally to discuss a peculiar observation of the pulpal floor.

## 2. Case Report

**Figure 1:**
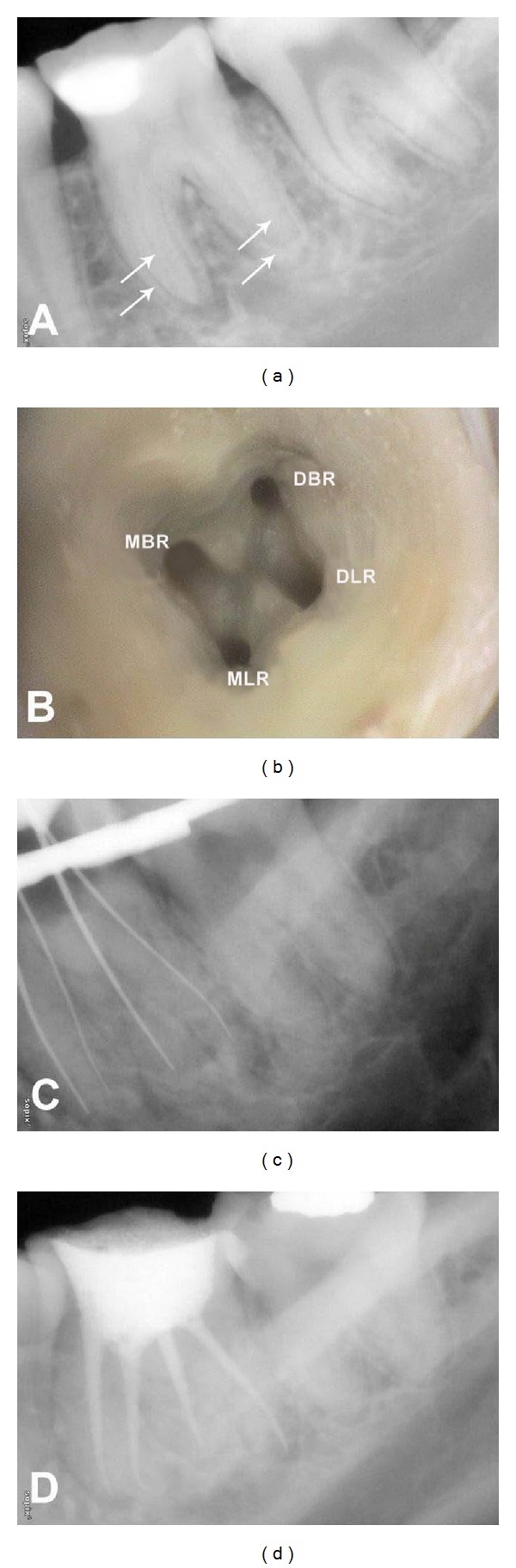
(a) Preoperative radiograph of tooth 19. (b) Access opening showing four canals (MBR: Mesiobuccal; MLR: Mesiolingual; DBR: Distobuccal; DLR: Distolingual). (c) Working length radiograph of tooth 19 in eccentric angulation. (d) Postobturation radiograph.

A 27-year-old female reported with the chief complaint of a “fractured filling” in her left lower back tooth. History revealed intermittent pain localized to the same tooth during mastication. The tooth was previously restored with a silver amalgam restoration 7 years back. Clinical examination revealed a silver amalgam restoration with secondary caries in the left mandibular first molar (tooth 19) which was tender to percussion. Vitality tests elicited no response. Preoperative radiographs showed widening of periodontal ligament space in relation to the mesial root apex. In addition, radiographic apical contour of the tooth suggested that there might be two distal and two mesial roots (Figure 1(a)). From the clinical and radiographic findings a diagnosis of pulpal necrosis with symptomatic apical periodontitis was made and endodontic treatment was initiated.

 Following endodontic access cavity preparation, two mesial and one buccally placed distal canal orifice was identified. Upon visual inspection of the floor of the pulp chamber using a dental operating microscope, a dark line was observed extending from the distal canal orifice towards the distolingual corner. At this corner, the overlying dentin was removed and a second distal canal orifice was detected. The conventional access was modified to improve access to the additional canals (Figure 1(b)). Root canal orifices were named as per the nomenclature proposed by Albuquerque et al. [8]. Working length was confirmed (Figure 1(c)) and the canals were instrumented. Calcium hydroxide was placed as an intracanal medicament with a lentulospiral and the access cavity was sealed with Cavit G. The patient was asymptomatic at the next appointment, a week later, which allowed for root canal obturation and a coronal composite restoration (Figure 1(d)). 

## 3. Discussion

A number of anatomical variations have been described in the mandibular first molar. Kottoor et al. [9] reported the presence of three distal canals, while Ghoddusi et al. [10] noted the presence of four distal canals. Presence of three [11] and four [5] mesial root canals has also been reported. Like the number of root canals, the number of roots may also vary. The major variant in this tooth type is the presence of an additional third root; a supernumerary distolingual root called radix entomolaris. Its prevalence varies in different populations ranging from 3% of the African population [12] to more than 30% of the Mongoloid population [13]. An extremely rare variation of an additional mesiobuccal root is called the radix paramolaris (RP) [14]. 

Morita [15] in a laboratory study examined 2,164 extracted mandibular first molars. He reported only a single four-rooted first molar, in a male patient, which formed 0.04% of the total sampled Japanese population (Mongoloid race). Various other extensive laboratory studies in different population and ethnic groups have not reported a single four-rooted mandibular first molar [16–18]. Only two case reports have previously described the presence of four-rooted mandibular first molar [19, 20]. However, both have reported three distal and one mesial root. The present report describes a four-rooted mandibular first molar with two mesial and two distal roots in which each of the four roots have an independent root canal.

**Figure 2:**
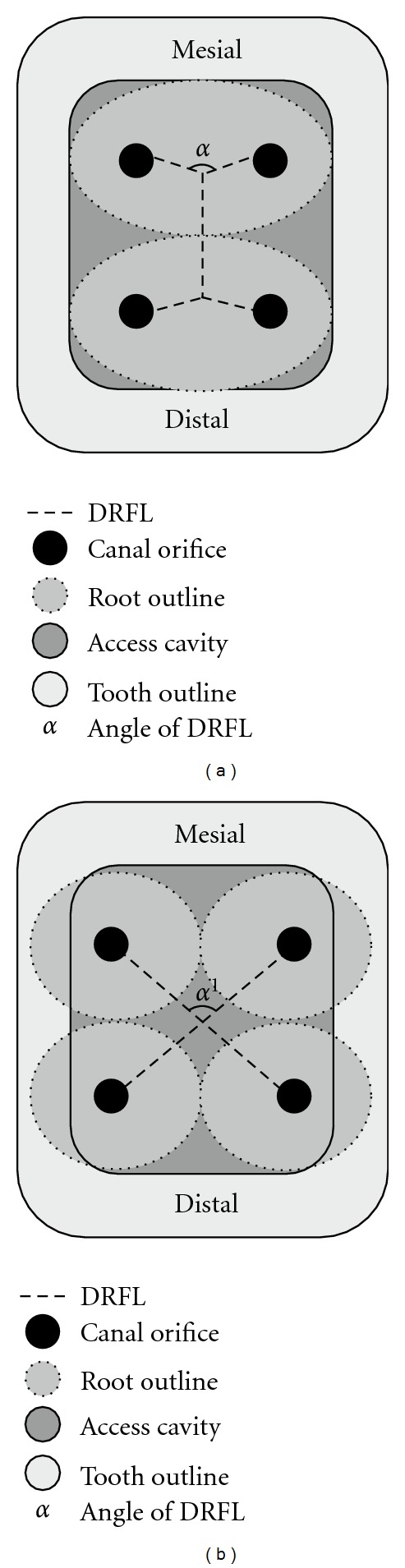
Diagrammatic representations illustrating the correlation between the developmental root fusion lines (DRFL) on the pulpal floor with the canal orifices in permanent mandibular first molars with varying root and root canal anatomy. (a) A more obtuse angle (*α*) between the connecting DRFL in teeth with 2 roots, 4 canals. (b) A more acute angle (*α* ^1^) between the connecting DRFL in teeth with 4 roots, 4 canals.

In mandibular first molars with two roots and each root having two distinct canals (for instance, mesiobuccal and mesiolingual canals in the mesial root), the angle formed between the developmental root fusion lines (DRFLs) joining these canal orifices is more obtuse [21]. This can be visualized in both the mesial and distal DRFLs of such two-rooted mandibular first molars (Figure 2(a)). In the case presented here, the angle formed between the DRFLs joining both the mesial and distal orifices was more acute; presenting in the form of a letter “X” (Figure 1(b)). This could signify that when an additional root is present both mesially and distally, the angle between the DRFLs which connect the orifices changes to a more acute angle (Figure 2(b)). Thus, the relationships of the DRFLs joining the canal orifices could provide an insight into the root anatomy of mandibular first molars. Further clarification of these observations would be required by individual laboratory studies or other case reports to allow their use as diagnostic criteria in cases of four-rooted mandibular first molars wherein each root presents with a single canal.

## 4. Conclusion

Successful endodontic treatment begins with proper clinical and radiographic examinations. A practitioner must be vigilant, as variations of root and canal anatomy might be encountered at any time during treatment. This paper may intensify the complexity of mandibular first molar variation and is intended to reinforce clinicians' awareness of the variable morphology of root canals.
